# Laboratory methods for case finding in human psittacosis outbreaks: a systematic review

**DOI:** 10.1186/s12879-018-3317-0

**Published:** 2018-08-30

**Authors:** Annelies A. Nieuwenhuizen, Frederika Dijkstra, Daan W. Notermans, Wim van der Hoek

**Affiliations:** 1 Centre for Infectious Diseases, Epidemiology and Surveillance, Centre for Infectious Disease Control, National Institute for Public Health and the Environment (RIVM), Bilthoven, The Netherlands; 2 Centre for Infectious Diseases Research, Diagnostics and Laboratory Surveillance, Centre for Infectious Disease Control, National Institute for Public Health and the Environment (RIVM), Bilthoven, The Netherlands

**Keywords:** Psittacosis, Chlamydia psittaci, Diagnostics, Disease outbreaks, Epidemiology, Systematic review, Zoonoses.

## Abstract

**Background:**

Psittacosis outbreak investigations require rapid identification of cases in order to trace possible sources and perform public health risk assessments. In recent outbreaks in the Netherlands, such investigations were hampered by the non-specificity of laboratory testing methods to identify human *Chlamydia psittaci* infections.

**Method:**

A systematic search of PubMed and Scopus databases of literature published between 01 January, 1986 and 03 July, 2017 was done to find best practices of laboratory-testing methods used in psittacosis outbreaks of two or more human cases. Reference lists of included articles were hand searched to identify additional articles.

**Results:**

Thirty-seven eligible articles were identified, describing 44 human psittacosis outbreaks in 12 countries. Laboratory tests performed were PCR (with various targets), serologic tests (complement binding reactions, ELISA’s, immunofluorescence tests and immuno-peroxidase tests) and culture, in various combinations. The literature provided no ‘gold standard’ laboratory testing strategy to identify recent human *C. psittaci* infections. In most psittacosis outbreaks, for a considerable number of cases (or tested individuals in an exposed cohort), *C. psittaci* infection could not be confirmed, nor excluded as causative pathogen. None of the testing strategies was found to be suitable for (nearly) full case finding.

**Conclusion:**

PCR enables rapid identification of human psittacosis patients and helps source finding by genotyping but has the disadvantage that sensitivity is high only in the acute phase. In outbreak situations, there is often a time delay and therefore, there is a need for new serologic testing methods next to PCR, with good specificity and sensitivity. Moreover, serum is easier to collect than the preferred diagnostic materials for PCR. A serologic test that can reliably confirm infection status without the necessity of convalescent serum sampling would enhance case finding, source tracing, identification of risk factors and assessment of burden of disease in various settings.

**Electronic supplementary material:**

The online version of this article (10.1186/s12879-018-3317-0) contains supplementary material, which is available to authorized users.

## Background

Psittacosis is a zoonotic disease, which regularly causes small outbreaks worldwide. Traditionally, psittacines (parrot-type birds) have been considered as reservoir of the causative bacterium, *Chlamydia psittaci*. In addition, many other birds, including wild birds and commercially kept poultry, have also been implicated [1].

In the Netherlands, an outbreak occurred in 2007 among visitors to a bird show [2, 3]. After confirmation of the diagnosis by real-time polymerase chain reaction (PCR) of three hospitalized patients, a retrospective cohort study among about 200 visitors was started. Serological screening and a questionnaire study were performed in order to estimate attack rates and identify risk factors. Based on screening immunoglobulins (Ig)(IgG/A/M) with a genus specific enzyme-linked immunosorbent assay (Chlamydia r-ELISA, Medac Diagnostika) and a complement fixation test (CFT) on sera taken 23 days after the bird show, the attack rate was very high (42/156, 27%), but surprisingly none of the IgM concentrations of suspected infected visitors showed a significant rise after a mean of 14 days to confirm a recent infection. To verify the unexpected high attack rate, a set of control sera of 30 healthy volunteers was also tested with the same ELISA. Thirty percent of the single control sera showed an IgM response. None of these serological responses could be explained by zoonotic bird contact. It was unclear if this high seroprevalence was due to cross-reaction with other *Chlamydia* species, especially with *Chlamydia pneumoniae* or false positivity of the ELISA test [2, 4, 5]. The lack of a *C. psittaci*-specific antibody test therefore hampered a proper serological interpretation of the epidemiological outbreak investigation.

In 2012, similar problems occurred with investigation of another outbreak in the Netherlands with eight confirmed cases among visitors and volunteers working for a bird sanctuary. Three persons were hospitalized and diagnosed with pneumonia (including the index case). For the epidemiologic investigation of this outbreak, a cohort of more than a 100 volunteers as well as 3 payed workers was requested to fill in a questionnaire about demographics, symptoms, medical care sought, medication use, medical history and possible exposures. Out of 40 respondents who reported symptoms, 25 met the formulated clinical case definition for psittacosis of this outbreak. Convalescent serological samples of 19 cases were taken three times within a 3–4 week interval. Serology was performed on these samples for the identification of antibodies against *C. psittaci* (micro-immunofluorescence tests (MIF) IgG for *C. psittaci)* and to exclude other *Chlamydia* species (MIF IgG for *C. pneumoniae*, ELISA IgM/A/G for *C. pneumoniae* and MIF IgG for *C. trachomatis*). Six patients tested positive for *C. psittaci* with a fourfold rise in IgG titer. PCR for *C. psittaci* was used in 12 patients, of whom two tested positive. Nasal-pharyngeal swabs for PCR testing were taken three to 14 days after onset of symptoms with exception of a sputum sample of an intensive care (ICU) patient, which was taken after 22 days. PCR to exclude *C. pneumoniae*, *Coxiella burnetii*, and influenza virus A was performed in nine patients, who all tested negative for these pathogens [6](personal communication N. Reedijk 17–08-2016 and 21–03-2017).

This meant that despite the use of a combination of laboratory tests, *C. psittaci* could be confirmed in a small number of the suspected cases only. For majority of the cases tested, it remained unclear whether infection with *C. psittaci* was the cause of their symptoms. Therefore, in spite of the availability of extensive information from the questionnaire, formal epidemiological analysis lacked possibilities and was unsatisfactory. The difficulties with the laboratory diagnostics in this outbreak were amongst others related to omitting laboratory diagnostics for *C. psittaci* by physicians (patients are treated empirically), non-optimal sampling intervals (caused by medical consultation delay and sampling delay) and lack of suitable clinical material for PCR testing (no sputum or broncho-alveolar lavage (BAL) available for non-hospitalized patients) [4, 6], (personal communication N. Reedijk 17–08-2016).

Both outbreaks in the Netherlands showed the constraints in confirming human psittacosis cases with PCR-based diagnostics because of time delay, decline of sensitivity of PCR in time and/or unavailability of appropriate diagnostic material. Serology with convalescent sampling is the alternative to screen possible exposed persons.

The difficulties in interpreting laboratory findings in these outbreak settings prompted us to do a systematic review of the international literature on psittacosis outbreaks with special emphasis on the laboratory methods used, in order to find out which (combination of) laboratory testing methods could be advised for psittacosis outbreak investigations.

## Methods

### Search strategy

The search strategy we developed aimed to find descriptions of human psittacosis outbreaks with a special focus on diagnostic laboratory methods. We searched PubMed and Scopus for items published between 1 January 1986 and 3 July 2017, using MESH (Medical Subject Headings)and keywords psittacosis, *Chlamydia* or *Chlamydophila psittaci*, psittaci, outbreak*, disease outbreaks, epidemiology, epidemic, human(s) and not animals. The complete search strategies are given in Additional file 1. All results were combined in one EndNote X8 file (Clarivate Analytics USA) and duplicates removed using EndNote and by hand. There was no language restriction in our search but we could only read full texts of selected articles in Dutch, English, German, French and Spanish. At a later stage, reference lists of selected full texts were checked to identify studies possibly missed by our search strategy. Finally, the Cochrane library was checked for systematic reviews that included information on the topic of our review with the keyword ‘psittacosis’ and keywords ‘*Chlamydia psittaci*’, without relevant results.

### In- and exclusion criteria

**Fig. 1:**
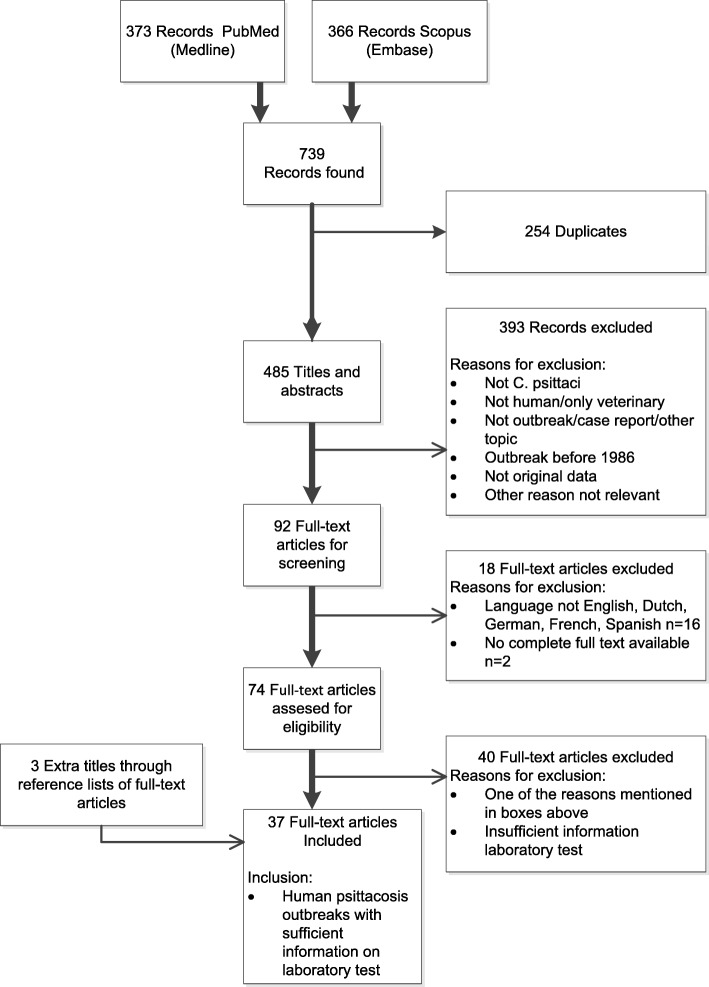
PRISMA flow diagram of the systematic search and selection results

Two authors (AN, FD) used a pilot search of PubMed with keywords ‘psittacosis outbreak’ to specify in- and exclusion criteria before the title and abstract screening. Figure 1 shows the definitive in- and exclusion criteria. In summary, articles describing an outbreak of human psittacosis with at least two possible human cases and with specification of the laboratory test methods used to confirm infection were included. Articles with description of psittacosis outbreaks among humans that took place before 1986 were excluded, because before that time, *C. pneumoniae* was designated as *C. psittaci* strain TWAR (Taiwan acute respiratory agent) [7, 8] and could not be differentiated from *C. psittaci* [9, 10]. We also excluded single case reports and reviews or other publications not based on original data. We did not find any human outbreaks when articles mentioned *C. psittaci* as cause of abortion, ocular lymphoma or trachoma in the pilot search. Articles with these topics were therefore other reasons for exclusion. Full text articles, in which the type of laboratory tests that were used were not specified, were also excluded.

### Title and abstract screening

In the first screening stage, the in- and exclusion criteria were applied to the titles and abstracts resulting from the literature search. This title and abstract screening was done independently by two investigators (AN, FD) for the PubMed and Scopus search results. PubMed and Scopus items were excluded when both authors considered them not relevant. An item was selected from PubMed or Scopus search for full text screening if at least one of the authors (AN, FD, WvdH) labeled it as possibly meeting the inclusion criteria. Articles without abstract were included for full text screening when the title seemed relevant. When at least one of the authors (AN/FD, AN/WvdH) had doubt about exclusion after the initial selection round of PubMed or Scopus database items for full text screening eligibility, a third author was consulted and disagreements solved by discussion.

### Full text screening

In the second screening stage, the in- and exclusion criteria were applied to the full text articles that resulted from the title and abstract screening. Three authors (AN, FD, WvdH) did this independently. Disagreements about selecting an item were resolved by discussion between the authors (AN, FD, WvdH). This yielded the full text articles that were included in the review.

Reference lists of the selected full text were checked by hand to find extra titles that were missed by the search. These extra titles were screened for inclusion by the same method as the PubMed and Scopus titles.

### Data extraction

We extracted the following data from the included articles: year and country of the outbreak, population and setting, the laboratory test(s) performed to diagnose *C. psittaci* infection in humans, number of patients tested as well as number of patients positive by the laboratory test. Data extraction was done by AN. Uncertainties were resolved by discussion between the authors (AN, FD, WvdH).

## Results

### Characteristics of articles included for review

Our search strategy resulted in 739 titles of which 34 articles met our criteria for full text inclusion for review (see Fig. 1). The PubMed (373) and Scopus (366) database provided 34 of these articles. In addition, three more articles were found by checking the reference lists of the included full texts. The selected 37 eligible articles described 44 outbreaks of human psittacosis in twelve different countries over the period 1986 through 2014 (Table 1). Seven of the 16 larger outbreaks with ten or more cases, took place in poultry processing plants/slaughterhouses and poultry farms [11–17]. Other larger outbreaks were related to a bird show or bird park [3, 18, 19], at a veterinary teaching hospital [20], an aviary in an institution [21], a distribution of birds from the same breeder [22] and two were linked to wild birds in Australia [23, 24]. In one large outbreak transmission took place in hospital setting [25]. More than half of the articles (21/37, 57%) described one or more smaller outbreaks with two to nine people tested with laboratory methods. Eight [26–33] of these smaller outbreaks occurred at family homes and were linked to pet birds, six took place at poultry farms or poultry processing plants [31, 34–38], three were linked to a (pet or other) shop [39–41], two described outbreaks at hospitals, with human psittacosis patients as source [42, 43], two concerned workers and students at a veterinary clinic or school [44, 45] and one was linked to a parrot relief and breeding center [46].

**Table 1:** Included studies of human psittacosis outbreaks and the laboratory testing methods used for *Chlamydia psittaci*

Article, publication year[reference]	Outbreak year, country	Population, setting	Laboratory test *C. psittaci*, numbers positive/numbers tested			Remarks
			PCR	Serology (name test, numbers)	Culture	
Huminer et al., 1988 [31]	1986–1987, Israel	8 Families in contact with pet birds or domestic fowl	No	IPA 24/36 with 8/36 seroconversion IgM	Throat swab 22/35	8 Family outbreaks with exclusion of *C. pneumoniae* by MIF; 1 case possible person-to-person transmission; 3 outbreaks with sick household birds (parrots) as index case.
Bourke et al., 1989 [27]	NM, UK	Family with aviary outside house	No	CFT 3/9 and MIF 3/9	Nasopharyngeal aspirates, after antibiotics 0/3(?)	Serological cross-reactions with *C. trachomatis* and *C. pneumoniae* gave difficulties.
CDC, 1990 [11]	1989, USA	Workers turkey processing plant	No	CFT 40/60	BAL specimen, 1 (seropositive) case	24/40 Hospitalized.
Samra et al., 1991 [32]	1987, Israel	Family owning 3 parrots of which 2 recently died	No	IPA (IgM, IgG, IgA,) 4/4	Saliva, throat, sputum 3/4	2/4 Patients developed hepatitis.
Morrison et al., 1991 [39]	1989, UK	Employees and visitors pet shop, after delivery new *Agapornis* birds	No	CFT 7/7	No	2 Patients died.
CDC, 1992 [26]	1992, USA	2 Families bought pet birds (parakeet, cockatiel) linked to bird distributor	No	CFT family A 1/1, family B 2/6	No	Child and mother of family A mucopurulent conjunctivitis.
Hedberg et al., 1989 [12]	1986, USA	Workers at turkey farm and processing plants	No	CFT 122/697 workers; 122/186 suspected cases	No	13 Hospitalized.
Hinton et al., 1993 [13]	1989–1990, Australia	Workers duck farm and processing plant	No	CFT 19/25 with 3/19 seroconversion or IgM positive	No	3 Symptomatic cases; Significant association length of employment and presence of antibodies; Isolation of *C. psittaci* in birds.
Schlossberg et al., 1993 [21]	1991–1992, USA	Contacts private aviary in basement and outdoors with poultry, psittacine birds, canaries and finches	No	CFT 13/24 (1 confirmed, 10 presumptive, 2 suspected cases)	No	Joint swelling 3 cases, 1 case had a rash and 3 seropositive cases without symptoms.
Davies et al., 1995 [28]	NM, UK	Cases associated with 2 linked aviaries(e.g. parrots, cockatiels, parakeets)	No	CFT 9/12, IF or CFT positives 9/9	No	Bird contact reported by 5/9 cases. 1 Fatal case. IF to exclude *C. pneumoniae*.
Hughes et al., 1997 [42]	1994, USA	Hospital staff and roommate exposed to pet-shop worker with psittacosis	No	MIF IgM, IgG 5/8	?	Person-to-person transmission likely; Cross-reactivity possible as 3 cases were also MIF seropositive for *C. pneumoniae.*
Goupil et al., 1998 [14]	1990, France	Workers poultry slaughterhouse, truck driver, maintenance mechanic	No	CFT or IF seroconversion with 5 week interval 18/56	No	Retrospective study symptomatic workers of whom 6 pneumonia and 4 hospitalized.
Moroney et al., 1998 [22]	1995, USA	Owners of recently bought birds originating from 1 breeder (parakeets, finches, cockatiels, love birds, conures, canaries, parrots)	No	MIF 10/58	No	Human outbreak identified after diagnosis of chlamydiosis in birds. Clinical illness did not correspond with serological response.
Williams et al., 1998 [23]	1995, Australia	People living close to forest with many native parrots	Post-mortem lung tissue 1/1	IF screening 16 positive; CFT 14/16 (four-fold rise or seroconversion)	No	Case-control study; < 2% seropositivity among controls; 1 person died possibly infected by person-to-person transmission. Comment in [59, 60].
Lederer et al., 1999 [15]	NM, Germany	Comparison of (a) 82 workers chicken, duck, and geese processing plant of whom 8 symptomatic with; (b) 83 workers other chicken processing plant and (c) 82 non-occupationally exposed	No	(a) CFT/IPA 57/82 and MIF 13/25 (b) CFT/IPA 16/83 and MIF 0/8; (c) CFT/IPA 22/82 and MIF 0/17	No	10/18 Workers employed < 3 months had serological signs of recent infection. Two cases died of multi-organ failure, 6 others had pneumonia. Also tested for *C. pneumoniae* and *C. trachomatis* in MIF.
Ito et al., 2002 [41]	NM, Japan	Family, index case visited confectionary with parakeets	No	CFT and MIF 2/2	?	Possible person-to-person transmission between sisters. Index case rising CFT titer, sister static high CFT titer. *C. pneumoniae* and *C. trachomatis* checked by MIF.
Telfer et al., 2005 [24]	2002, Australia	Residents with direct or indirect wild bird contact (parrots, doves, currawongs, magpies)	(Only for exclusion *C. pneumoniae* 0/8)	MIF 59/95 with 35/59 seroconversion or 4-fold titer rise; 33/35 also 4-fold rise CFT; 3 MIF negatives had 4-fold rise in CFT	No	Case-control study. 2 ICU cases. Probable source wild birds in yards; 1 king parrot tested *C. psittaci* positive. MIF/EIA for exclusion *C. trachomatis* and MIF *C. pneumoniae*. No evidence of infection by other respiratory pathogens in probable *C. psittaci* cases.
Saito et al., 2005 [40]	NM, Japan	Owner and co-worker of pet shop selling parrots, parakeets and budgerigars	No	CFT 2/2; MIF 2/2 (seroconversion)	?	Cross-reactivity with ELISA for *C. pneumoniae*. Infection by *Chlamydophilia avium* in a couple working in a pet shop. Comment about nomenclature *Chlamydophilia avium* [61]
Heddema et al., 2006 [20]	2004, Netherlands	Students and staff veterinary teaching hospital in contact with cockatiels, Amazon parrots and pigeons	OmpA RT-PCR 6/29 (sputum, throat swabs)	R-ELISA 9/29, CFT on r-ELISA positive sera 6/9	No	3 Hospitalized cases including 1 ICU. Not all PCR positives also seropositive or vice versa. Genotype of *C. psittaci* PCR positive parrots matched with human cases.
Kaibu et al., 2006 [30]	2005, Japan	Family; index patient bought cockatiel parakeet from pet shop	Pharyngeal swab, 0/2	MIF four-fold rise 4/4	Pharyngeal swab 0/2	Dead bird *C. psittaci* PCR positive. *C. pneumoniae* and *C. trachomatis* also tested by MIF.
Harkinezhad et al., 2007 [46]	NM, Belgium	Veterinarian and veterinary assistant, tested after visiting parrot relief/breeding center, and manager of this center	OmpA nested PCR 3/3, genotype-spec RT-PCR 2/3 pharyngeal, nasal swabs	ELISA 2/2	Pharyngeal, nasal swabs 2/3	First report of transmission of genotype E/B from African grey parrots to humans. Veterinarian and assistant had only mild or no clinical signs.
Tiong et al., 2007 [16]	2003–2004, Australia	Workers duck abattoir and farm with ducks in open sheds in contact with wild birds	No	ELISA 53/97; IF 35/53	No	44/53 Cases reported symptoms and 5/12 pneumonia cases hospitalized. IF to exclude *C. pneumoniae* and other *Chlamydiae* spp. cross-reactions. 18/53 Significant cross-reaction to *C. pneumoniae.*
Berk, 2008 et al., [3]	2007, Netherlands	Visitors, participants bird show with many different bird species	RT-PCR 3/11 (2/11 throat swab, 1/5 sputum, 0/7 urine, 0/11 serum)	CFT 0/11 initial sample but seroconversion 9/11	No	23/> 200 Visitors ill and 11 hospitalized. Many CFT positives were not PCR positive. Bird source was a Siskin (not a psittaciformes).
Branley et al., 2008 [44]	2005, Australia	Staff veterinary surgery handling sick, wild psittacine bird	Spp. spec PCR (genus spec PCR) throat swab 1/3 (1/3), blood 1/3 (1/3), urine 0/3 (2/3)	MIF IgG 3/3	0/3	First report of PCR analyses on human blood and urine samples for diagnosing psittacosis and first report of using PCR for comparison organism load in human psittacosis patients compared to that of sick source bird. Wild bird culture positive. MIF and PCR (urine) negative for *C. trachomatis* (0/3).
Çiftçi et al., 2008 [29]	NM, Turkey	Family, bought 2 parrots a month ago	No	MIF 2/2	No?	First report of psittacosis from Turkey.
Gaede et al., 2008 [17]	2005, Germany	Veterinary officer, poultry breeders at infected poultry farm	RT-PCR and DNA micro-array assay BAL 2/2 and urine 1/1	CFT 111 samples of 65 ‘contacts’ – data not clear. 7 hospitalized cases positive. All CFT positive also MIF positive	No	17/24 Suspected human cases and 7 hospitalized confirmed cases, of whom 3 admitted to ICU; 1 patient died. *C. pneumoniae* tested by MIF. Birds PCR positive.
Matsui et al., 2008 [18]	2001–2002, Japan	Staff, students and visitors of bird park with hothouses	No	CFT 3/3 and MIF 14/14 symptomatic cases; MIF 8/91 non-symptomatic staff members	No	13/17 Hospitalized cases. Cross-reactions *C. pneumoniae* and *C. trachomatis* excluded by MIF.
Verminnen et al., 2008 [36]	2005–2006, Belgium	Turkey farmer and 2 scientists at turkey farm	Nested PCR 3/3 (sputum, pharyngeal, nasal swabs)	MIF 0/3 r-ELISA 3/3	3/3	Environmental monitoring study, not strictly an outbreak study. In air samples, chlamydial organisms detected by nested PCR. PCR *C. pneumoniae* and *C. trachomatis* negative.
Laroucau et al., 2009 (1) [37]	2006, France	Workers at duck farms and wife of worker	PCR 3/4 (1 BAL, 2 tracheal aspirates, 0 throat swab)	MIF 5/5	Tracheal aspirates 1/4; BAL, throat swab negative	5 Severe psittacosis cases but avian flu was suspected at first. Ducks and human cases linked by PCR sequencing OmpA gene. Cross-reactions *C. pneumoniae* and *C. trachomatis* checked by MIF.
Belchior et al., 2011 [19]	2008, France	Participants bird fair organized by breeders of *Psittacidae*	2 RT-PCR 2/3 throat swab	MIF 2/29 (probable cases)	No	Retrospective cohort (*n* = 86) study, AR 33/86, 11/48 suspected cases hospitalized, 29/48 tested by serology.
Yang et al., 2011 [38]	2009, China	Peacock farmers of a flock of sick peacocks	Genotyping OmpA on isolate	IF IgG 4/4 20 days post infection	Throat swab 1/4	First report of psittacosis in peacocks and peacock farmers.
McGuigan et al., 2012 [43]	2011–2012, Scotland (UK)	Outbreak investigation after pneumonia in 4 family members and 1 health care worker	RT-PCR 3/6, spp. specific PCR 3/3	CFT positive in 3 confirmed cases, 1 probable case and 4 possible cases	No	Person-to-person spread to healthcare worker with same PCR OmpA strain as index case and to 1 possible case staying at ICU with index case. Suspected *C. pneumoniae* outbreak turned out to be *C. psittaci* outbreak after PCR specification (spec RT-PCR *C. pneumoniae* 0/3).
Williams et al., 2013 [34]	2008, England (UK)	Poultry (mainly ducks) processing plant production line workers, engineering staff and visiting administrator	DNA microarray genotyping on DIF positive sputum sample 1/1	(Paired) CFT and WHIF; WHIF 7/9, 5/7 WHIF positive, 4/9 WHIF recent or rising CFT	No	2/3 Hospitalized cases ICU. AR 4% (9/225); 16/63 Persons some evidence of *C. psittaci* infection, 9/63 cases met case definition, 6/9 were symptomatic and sputum of 4/9 cases tested by DIF; 4/4 DIF positive. Cases only in plant processing free-range ducks.
Wallensten et al., 2014 [25]	2013, Sweden	Hospitalized psittacosis patient who transmitted the bacterium to family, hospital room mate and medical staff	PCR OmpA 3/3 (BAL)	MIF 4/11 IgM, 6/11 IgG, convalescent 1/6 IgM, 3/6 IgG	No	Human-to-human transmission proven and index ICU patient died. His wife and 5 other secondary cases needed hospitalization: 7 confirmed, 3 probable, 1 possible case; MIF negative for *C. trachomatis* and *C. pneumonia*
Laroucau et al., 2015 [35]	2013, France	Women with chicken gutting activities on a mixed poultry farm	RT-PCR Chlamydiaceae-spec 23SrRna specific IncA 4/5 sputum, 0/4 throat swabs	MIF 2/8	No	8 Hospitalized cases; throat swabs taken after start medication, 4 confirmed, 1 probable, 3 possible cases; in chickens *C. psittaci* and *C. gallinacea* detected
De Boeck et al., 2016 [33]	2013, Belgium	Belgian couple and daughter bought lovebird in pet shop in the Netherlands	Nested PCR OmpA 2/2, genotyping RT-PCR, pharyngeal swab	Indirect IF IgM 1/3, IgG 2/3	Pharyngeal swab 2/2	Couple hospitalized; daughter, tested after self-treatment. One patient seronegative but PCR positive. Birds and humans both genotype A
Chan et al., 2017 [45]	2014, Australia	People of veterinary school and horse stud farm in contact with fetal membrane of specific mare	No	EIA 3/3, MIF 0/3 4-fould rise, MIF 1/3 single high titer, MIF 2/3 falling titer	No	2 Hospitalized cases. AR 56%, 3 probable, 2 suspected cases of 9; mare serology EIA positive for Chlamydial spp. and fetal membrane qPCR positive; foal died 1 week old

Legend Table 1

*BAL* broncho-alveolar lavage, *CFT* complement fixation test, *DIF* direct immunofluorescence test, *EIA* enzyme immuno-assay, *ELISA* enzyme-linked immunosorbent assay, *r-ELISA* recombinant ELISA, *ICU* intensive care unit, *IF* immunofluorescence test, *IgA/IgM/IgG* immunoglobulin A/M/G, *IPA* indirect immunoperoxidase assay, *MIF* micro-immunofluorescence test, *NM* not mentioned, *OmpA* outer membrane protein, *PCR* polymerase chain reaction, *RT* real-time, *spp* species, *spec* specific, *WHIF* whole cell immunofluorescence test; ? data not clear

Psittacine birds were mentioned as possible sources in 43% (16/37) of the articles. Fifty-four percent (20/37) was linked to other birds of which 65% (13/20) to poultry. The articles of outbreaks at farms, breeders or processing plants all mentioned poultry, including chicken, ducks, geese, turkeys and peacocks, as possible source. In 16% (6/37) of the included articles direct or indirect contact with wild birds (for example: psittacines, pigeons, gulls, wild bird feathers or excrements), were considered possible sources [16, 23–25, 43, 44]. Exposure to an equine fetal membrane caused a small outbreak at a veterinary school and equine stud farm [45]. Some outbreaks occurred after hospitalization of a bird-infected index case, which spread the infection person-to-person amongst their contacts [25, 43].

### Laboratory tests used

In the included psittacosis outbreaks, culture, serology and PCR were the types of laboratory procedures that were performed to diagnose psittacosis or to exclude pathogens other than *C. psittaci* (e.g. *Legionella*, *Mycoplasma*) or cross reactivity (e.g., *C. pneumoniae*, *C. trachomatis*) (Table 1). Most articles describe combinations of these tests but in seven articles, all published before 1998, only one test was used to diagnose *C. psittaci* infection.

### Serology

In all of the included articles, at least one serological test was used. These were complement fixation tests (CFT), enzyme(−linked) immunosorbent assay tests (ELISA/EIA including recombinant ELISA), (micro-)immunofluorescence tests and whole cell immunofluorescence tests (IF/MIF/WHIF) and immuno-peroxidase tests (IPA). IF/MIF (including IPA [15, 31, 32] and WHIF [34]) was used in the majority of the articles (27/37, 73%), followed by CFT (20/37, 54%) and EIA/ELISA testing (5/37, 14%). CFT and IF were used regularly in outbreak investigations from 1985 up to 2014 in contrast to ELISA/EIA. Only in the period 2003–2007 [16, 20, 36, 46] and thereafter in 2014 [45] ELISA/EIA testing was found in our review.

In many articles problems occurred with the (un-)availability of convalescent serum samples needed for confirmation of cases or the second sample did not show seroconversion, which makes interpretation and conclusions difficult. Clinical symptoms, in combination with suspected test results, made assumption of a positive psittacosis infection case more acceptable for positive interpretation.

### PCR

PCR for laboratory diagnosis of *C. psittaci* was reported in 16 articles (16/37, 43%). In 2006, Heddema et al. were the first to describe PCR to identify *C. psittaci* in humans in an outbreak situation in 2004, although Williams et al. (1998) performed PCR on human lung tissue postmortem of one single case in 1995 [20, 23]. Telfer et al. (2005) used a *C. pneumoniae*-specific PCR on hospitalized case-patients to exclude *C. pneumoniae* in an outbreak in Australia in 2002 [24]. From 2006, PCR techniques to identify *C. psittaci* in human patients were reported in 15 of 19 (79%) of the included articles. PCR was used only in addition to a serologic testing method, while culture was used next to PCR in seven of the 15 PCR articles (47%). PCR was mainly performed on hospitalized patient samples. Among the articles, the PCR testing methods described differed in DNA targets, in species and genus specificity, and in amplification and detection techniques. In addition, there was variation in the sampled clinical material used for PCR testing. We found BAL, sputum, throat swabs, nasal and pharyngeal swabs, blood and urine as test material taken for PCR. BAL and sputum are considered the best material for performing *C. psittaci* PCR, but this was often not available. Easier to collect are nasal, pharyngeal and throat swabs, blood and urine. Verminnen et al. (2008) reported pharyngeal and nasal swabs to be more suitable than sputum but this could be due to problems to produce sputum in many patients [36]. Only in two studies more than 10 people were tested with PCR. Fifty percent (4/8) of the sputum samples and 14% (5/37) of the throat swabs tested PCR positive in these larger studies [3, 20]. For the other outbreaks, the numbers of cases tested with PCR were small. Throat swabs scored less frequently positive (17%, 8/48) compared to nasal and pharyngeal swabs, BAL and sputum. Nasal swabs scored best of the swabs (100%, *n* = 5) comparable with BAL (100%, *n* = 6) followed by sputum (71%, 12/17) and pharyngeal swabs (70%, 7/10). Urine was used for PCR in three outbreaks [3, 17, 44]. Gaede et al. (2008) reported *C. psittaci* genotype A detection in urine of a BAL positive patient [17]. Williams et al. (2013) used direct immunofluorescence test (DIF) on sputum samples and performed DNA micro-array based genotyping on one DIF positive sample [34].

**Fig. 2:**
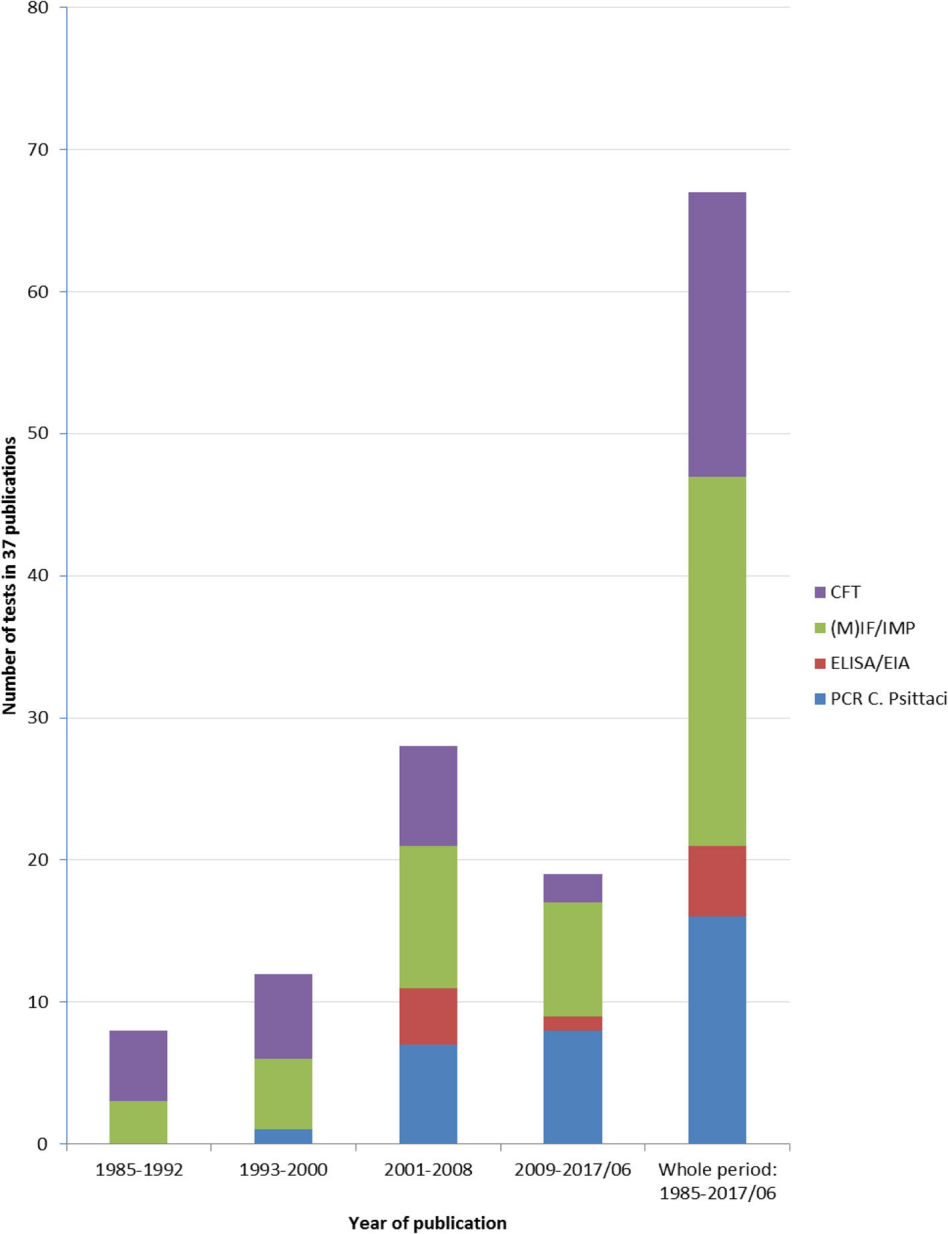
History of serology and PCR testing in psittacosis outbreaks 1985–2014. *1993–2000 PCR only on post-mortem material [34]

Figure 2 shows an overview of serology tests and PCR used in the psittacosis outbreaks of selected outbreak articles over time.

### Culture

Culture for *C. psittaci* was described in 30% (11/37) of all the articles, always in addition to other tests. Culturing *C. psittaci* was used mostly to confirm the outcome of the serological test(s) and/or PCR in one or a few index cases. Culturing other agents was done to be able to exclude these as causative pathogens. Culture was still used several times after PCR testing became available (58%, 7/12) (Table 1) although biosafety level three is needed and *C. psittaci* culture is not routinely performed in most diagnostic laboratories. BAL or sputum, pharyngeal aspirates or swabs are considered the best materials for culturing *C. psittaci,* and comparable to best material for PCR tests taken in the acute phase of infection.

In some articles included in the review that mentioned culture, it was not clear whether this was specifically for *C. psittaci*, or referred to routine (blood, sputum, urine) culture for other possible pathogens of respiratory infections. None of the blood cultures identified *C. psittaci*. This might indicate blood culture was performed mainly for other agents. Culturing of sputum (or other respiratory material) was more likely to include *C. psittaci* and more often reported positive results compared to blood cultures.

### Best practice

We did not find a standard algorithm or uniformity in testing methods. Wide varieties of testing methods and various sorts of combinations of these have been used. Each of these testing strategies was intended to deal with the low specificity and/or sensitivity of some of the individual tests and the availability of test material at the right moment during an infection period. A general tactic described in the articles in this review, both for PCR and for serology, is the use of a genus specific test as a first step, followed by a more specific (i.e. species-specific) test to exclude other chlamydial species, especially *C. pneumoniae* and *C. trachomatis*. Besides testing strategies to diagnose *C. psittaci*, many articles describe additional diagnostic tests to exclude other pathogens that may cause comparable clinical syndromes, especially influenza like illnesses and community acquired pneumonia (CAP). These tests may act as a first step in multi-stage testing. Testing for *C. psittaci* will be considered only after exclusion of those other pathogens, or when e.g. history of bird contact or other circumstances are suggestive for psittacosis. These first stage tests to exclude other pathogens are beyond the scope of this review.

Comprehensive testing strategies for outbreak situations were found in the articles of Heddema et al. (2006) [20] and of Belchior et al. (2011) [19]. In an outbreak at a veterinary teaching hospital, Heddema et al. (2006) used PCR [47] in as many subjects as possible in combination with serology (CFT and ELISA on paired samples) for human case finding. In addition, they performed PCR among potential bird sources for veterinary case finding and culture to exclude other human respiratory pathogens. In an outbreak study related to a bird show, Belchior et al. (2011) used serology (MIF) in combination with two different PCR tests (one targeting a *C. psittaci*-specific incA gene [48], and one targeting a Chlamydiaceae-specific 23S rRNA gene [49]) for human case finding. The latter PCR test was also used for veterinary source finding and genotypical matching but matching failed because genotyping of PCR positive human samples was impossible due to lack of sufficient DNA.

Both Heddema et al. (2006) and Belchior et al. (2011) were able to confirm *C. psittaci* as causative agent of the outbreak and exclude other chlamydial species. However, in both studies, only a few of the tested human cases could be confirmed despite early recognition of the outbreak. Most of the tested cases remained classified as ‘possible *C. psittaci’*. Nevertheless, with the testing strategy of Heddema et al. (2006), the investigators were able to identify the (possible) source of the outbreak by a genotypic match between a bird source and human cases.

## Discussion

In recent outbreaks in the Netherlands, serological laboratory test results to identify acute and non-acute human *C. psittaci* infections turned out to be non-specific, thereby precluding the monitoring of the number of confirmed cases, detection of possible sources, and identification of risk factors. The difficulties with laboratory testing strategies in outbreak settings prompted us to do a systematic review of the international literature of laboratory methods used in psittacosis outbreaks.

However, the main findings of the review are that a wide variety of (combinations of) testing methods are being used and that no (gold) standard or uniformity in testing strategies exists. For outbreak investigation, each single test method has drawbacks, ranging from low sensitivity or cross-reactivity with related species, to issues relating to the unfeasibility to collect the optimal clinical material at the optimal time intervals for the respective tests. It is clear that in a considerable number of cases (or tested individuals in an exposed cohort) in most psittacosis outbreaks, *C. psittaci* infection cannot be confirmed, nor can *C. psittaci* be excluded as causative pathogen. Although studies were found in which the investigators made considerable efforts to deal with known limitations of the single test methods, by collecting various sorts of clinical materials and using a broad combination of tests, none of these testing strategies seemed to be suitable for (nearly) full case finding.

### Evaluation of testing strategies

The current existing test methods for *C. psittaci* can be divided into culture, PCR and various serological methods, namely CFT, ELISA/EIA, IF and IPA tests. In all outbreaks, serologic methods were used. However, because of cross reactivity with other Chlamydial species, serological testing of a serum sample taken at one single moment will not be sufficient for confirmation [50]. Therefore, PCR testing or culture, which have a higher specificity, can be performed in addition. For these test methods, clinical material from the lower respiratory tract is preferred, i.e. sputum or BAL [51]. However, these materials are frequently not available, especially not in outpatients, and considered as rather invasive to obtain. PCR is also being performed on other clinical material, like throat swabs, but then sensitivity is assumed to be considerably lower although few comparative studies have been performed [36, 51, 52]. Furthermore, PCR is only of use early in the infection and is therefore of limited value for retrospective case finding or investigating asymptomatic exposure.

To overcome the problems with the low specificity of single point serology, serological testing on convalescent sera can be performed, taken with an interval of several weeks but we found no standard for the optimal time interval. This approach has two disadvantages. First, sera from the acute phase of the illness are necessary, which is only possible if all cases or cohort members are sampled early in the outbreak. Second, samples from a few weeks later are necessary. This requires a sampling procedure that might be difficult to implement in practice. Taking convalescent sera is time consuming, expensive and is unlikely to be relevant for clinical management of individual patients, whose symptoms might have already subsided after presumptive antibiotic therapy. At present time, ELISA techniques lack specificity and IF techniques are more reliable when they are performed in 3-spot IF, including *C. trachomatis, C. pneumoniae* and *C. psittaci.* Finding a high IgM titer, in combination with clinical symptoms and/or history of a patient, can provide an early probable case diagnosis before waiting for the second serum sample for confirmation.

In outbreaks of psittacosis, there is often a considerable time delay between onset of symptoms in patients and laboratory diagnostic testing. History of bird contact may be absent. When history is not taken carefully and bird contact unknown, standard diagnostics in patients with pneumonia usually do not include tests for *C. psittaci* infection [53]. Therefore, many psittacosis index patients are discovered late and in hospital situations. Or, they might not be recognised due to lack of sensitivity of PCR because test material is taken long after start of infection and/or first serum samples do not show high antibody titres. Diagnosis confirmation by serology takes at least two weeks after taking the first sample, and is therefore always retrospective. Culturing *C. psittaci* also takes more time than PCR for specific diagnosis, is not easy and has the disadvantage that it has to be performed under biosafety level three laboratory conditions. PCR is on the other hand, a specific and fast method when performed on suitable acute phase respiratory material. If the possibility exists to use a *C. psittaci* specific PCR (and not a genus specific PCR), this is the most specific and fastest method to confirm an individual suspected psittacosis patient. Nevertheless, the delays as mentioned above hamper rapid specific diagnosis of psittacosis especially by PCR when bird contact is not obvious. Of course, the chosen testing strategy will depend also on the availability of tests, test material and their sensitivity and specificity in combination with the likelihood of a psittacosis case or possible outbreak in mind.

### Implications for epidemiological investigation

In any psittacosis outbreak, confirmation of human cases will take some time, because of the incubation time of the disease, patient delay in seeking medical care, diagnostic delay (i.e. the time from first medical examination to a possible or confirmed diagnosis), and delay in recognition that there is a possible outbreak situation, after the first human and/or veterinary cases have occurred. In practice, this period might last several weeks. Therefore, delays in sampling of symptomatic cases and asymptomatic exposed people cannot be prevented. This means that full case finding with the current available laboratory methods for the diagnosis of *C. psittaci*, is impossible. Rather than trying to test all possible cases, it seems more efficient to focus on early confirmation of an outbreak based on PCR testing of only a few cases, i.e. confirm *C. psittaci* with laboratory methods in some epidemiological linked individuals. Genotyping of PCR-positive samples will facilitate source tracing. In addition, further risk factor analysis can then be based on a clinical case definition. However, with many probable and possible cases, based on such a clinical case definition, it will be difficult to obtain reliable estimates of disease burden.

### Limitations of the study

Our review is based on three decades of international literature on laboratory-testing strategies used for the diagnosis of *C. psittaci* in human psittacosis outbreaks. Unfortunately, we had to exclude 18 potential relevant articles because of the language or because we were not able to retrieve the full text. However, the English abstracts of these excluded articles, when available, did not describe deviating methods, settings or populations from the included literature.

Another difficulty in the selection process of articles was when more than one case was described and we had to consider if the setting was an outbreak or not. Some interesting articles had unusual situation. Vorimore et al. (2015) described several psittacosis cases but we considered these more likely to be separate cases gathered over time than to be part of an outbreak. These cases worked on different duck farms with laying flocks but had duck insemination with semen from a single male flock, diagnosed as heavy shedders, in common [54]. Branley et al. (2014) described an endemic situation in Australia of community-acquired psittacosis in the period 2003–2009 but not an outbreak. Sixty percent of the cases did not have history of direct bird contact but indirect contact was universal. Moreover, only a low prevalence of *C. pneumoniae* was present [55]. The systematic review is complicated by the changes in nomenclature over the years, from *Chlamydia* to *Chlamydophila psittaci* and back, and the discovery of new Chlamydia subtypes. We excluded newly described non-avian strains, such as *C. abortus*, *C. caviae*, *C. felis* and *C*. *pecorum,* by restricting our search to *C. psittaci* outbreaks with a link to birds only and not to other animals. Outbreaks of human psittacosis without bird contact, i.e. caused by non-avian strains, have not been reported, although case reports have been published [56]. Recently, two more Chlamydiae species were described to infect birds i.e. *C. avium* and *C. gallinacea*. The zoonotic potential of these new species is not established well yet, but infection may not be detected by the currently available PCR tests for *C. psittaci* [57, 58]. Laroucau et al. (2015) describe human psittacosis cases in contact with *C. gallinacea* and *C. psittaci* co-infected poultry but *C. gallinacea* could not be identified in the human cases [35].

Another challenge for the present review was that laboratory methods and case definitions in the included articles often were described poorly and differed between outbreaks. Therefore, it was difficult to categorize the reported laboratory methods and to assess the number of cases tested positive.

## Conclusion

PCR enables rapid identification of acute symptomatic and asymptomatic human psittacosis patients and helps source finding by genotyping in outbreaks. However, sensitivity of PCR declines rapidly in the time period between onset of illness and seeking medical care and performing laboratory testing. Moreover, suitable material to use for PCR testing is not easily available. We conclude that there is a need for new serologic testing methods next to PCR, with good specificity and sensitivity, preferably in a single sample, for confirmation of psittacosis cases in outbreaks.

## Additional file

Additional file 1: Search Strategies. (DOCX 14 kb)

## Abbreviations

AR: Attack Rate
BAL: Broncho-alveolar lavage
*C. abortus*: *Chlamydia abortus*
*C. avium*: *Chlamydia avium*
*C. caviae*: *Chlamydia caviae*
*C. felis*: *Chlamydia felis*
*C. gallinacea*: *Chlamydia gallinacea*
*C. pecorum*: *Chlamydia pecorum*
*C. pneumoniae*: *Chlamydia pneumoniae*
*C. psittaci*: *Chlamydia psittaci*
*C. trachomatis*: *Chlamydia trachomatis*
CFT: Complement fixation test
DIF: Direct immunofluorescence test
DNA: Deoxyribonucleic acid
EIA: Enzyme immuno-assay
ELISA: Enzyme-linked immunosorbent assay
ICU: Intensive care unit
IF: Immunofluorescence test
IgA/IgM/IgG: Immunoglobulin A/M/G
IPA: Indirect immunoperoxidase assay
MESH: Medical Subject Headings
MIF: Micro-immunofluorescence test
NM: Not mentioned
OmpA: Outer membrane protein
PCR: Polymerase chain reaction
PRISMA: Preferred Reporting Items for Systematic Reviews and Meta-Analysis
r-ELISA: Recombinant ELISA
RT: Real-time
spec: Specific
spp.: Species
TWAR: Taiwan acute respiratory agent
WHIF: Whole cell immunofluorescence test
