# Supplementary material for: Laboratory methods for case finding in human psittacosis outbreaks: a systematic review
Source: BMC Infect Dis. 2018 Aug 30;18:442. doi: 10.1186/s12879-018-3317-0 (PMC6118005; doi:10.1186/s12879-018-3317-0)
Supplement: Supplementary file 1 — Search Strategies. (DOCX 14 kb) [file 12879_2018_3317_MOESM1_ESM.docx]

**Additional file 1: Search strategies**

*Pilot search string PUBMED*:

(("psittacosis"[MeSH Terms] or "psittacosis"[All Fields]) and ("disease outbreaks"[MeSH Terms] or ("disease"[All Fields] and "outbreaks"[All Fields]) or "disease outbreaks"[All Fields] or "outbreak"[All Fields])) and ("1986/01/01"[PDAT] : "2012/08/17"[PDAT])

**PubMed search**:

(((psittacosis[mh] OR chlamydophila psittaci [mh] OR psittacosis[tiab] OR psittaci[tiab]) AND (outbreak*[tiab] OR epidem*[tiab] OR disease outbreaks[mh] OR epidemiology[sh] OR human[ti] OR humans[ti])) NOT (animals[mh] NOT humans[mh])) AND ("1986/01/01"[dp]:"2017/07/03"[dp])

**Scopus search**:

(((TITLE-ABS-KEY(psittacosis OR psittaci) AND (TITLE-ABS-KEY(outbreak* OR epidem*) OR TITLE(human OR humans))) AND NOT (KEY(animal OR animals OR animalia OR birds OR cattle OR nonhuman OR non-human) AND NOT KEY(human OR humans))) AND PUBYEAR > 1985

The latest search date was 3 July 2017.

The outcome of PubMed and Scopus was collected in EndnoteX8. The PubMed search Without epidemiology (sh) was used to compare and remove duplicates with the Scopus search with EndNoteX8. This combined Endnotefile was also checked for missed duplicates by hand, afterwards.
